# The end of a myth—Bt (Cry1Ab) maize does not harm green lacewings

**DOI:** 10.3389/fpls.2014.00391

**Published:** 2014-08-12

**Authors:** Jörg Romeis, Michael Meissle, Steven E. Naranjo, Yunhe Li, Franz Bigler

**Affiliations:** ^1^Agroscope, Institute for Sustainability Sciences ISSZurich, Switzerland; ^2^USDA-ARS, Arid Land Agricultural Research CenterMaricopa, AZ, USA; ^3^State Key Laboratory for Biology of Plant Diseases and Insect Pests, Institute of Plant Protection, Chinese Academy of Agricultural SciencesBeijing, China

**Keywords:** *Chrysoperla carnea*, environmental risk assessment, meta-analyses, non-target effects, study design

## Abstract

A concern with Bt-transgenic insect-resistant plants is their potential to harm non-target organisms. Early studies reported that Cry1Ab-producing Bt maize and purified Cry1Ab harmed larvae of the green lacewing, *Chrysoperla carnea*. Although these effects could not be confirmed in subsequent studies, some authors still refer to them as evidence that Bt maize harms beneficial species. We provide a comprehensive review of the studies evaluating the effects of Bt (Cry1Ab) maize on *C. carnea*. The evidence indicates that this important predator is not affected by Bt maize or by the produced Cry1Ab protein. We discuss how conceptual models can assist environmental risk assessments, and we emphasize the importance of robust and reproducible studies.

## Introduction

One concern with the growing of genetically modified (GM) maize that produces insecticidal Cry proteins from the bacterium *Bacillus thuringiensis* (so-called Bt maize, Box 1) is that the insecticidal proteins may harm non-target organisms that provide important ecosystem services including biological control, pollination, and decomposition. Organisms providing these services must be protected from unacceptable adverse effects of pest control measures, i.e., they are classified as “protection goals” (Nienstedt et al., 2012; Sanvido et al., 2012; Garcia-Alonso and Raybould, 2013). Effects on such protected entities are thus addressed in the environmental risk assessment that precedes the approval for cultivation of new GM plants (Garcia-Alonso et al., 2006; OECD, 2007; Rose, 2007; Romeis et al., 2008; Devos et al., 2014).

Box 1 **Bt maize.**

Genetically modified (GM) maize expressing the *cry1Ab* gene derived from the bacterium *Bacillus thuringiensis* (Bt) was first commercialized in 1996. In 2013, maize producing Cry1Ab was cultivated on millions of hectares in the Americas, South Africa, the Philippines, and Europe (mainly Spain) (James, 2013).

Bt maize containing Cry1Ab is protected from major lepidopteran pests such as the European corn borer (*Ostrinia nubilalis*; Crambidae), the Asian corn borer (*Ostrinia furnacalis*; Crambidae), and the Mediterranean corn borer (*Sesamia nonagrioides*; Noctuidae) (Hellmich et al., 2008).

At present, three transformation events that express *cry1Ab* have been developed (CERA, 2012). The most common event that is commercialized in the countries listed above is MON810. A second event, Bt11, is grown in most of those countries but has been waiting approval for cultivation in the European Union since 1996. The third event, Bt176, was cultivated from 1996 to 2007. In addition, stacked events that contain MON810 and/or Bt11 are cultivated or in the approval process (CERA, 2012; EFSA, 2013).

Plants carrying any of the three events do not differ greatly in the *cry1Ab* expression levels in green plant tissue (leaves). The amount of Cry1Ab in leaves of the different transformation events typically ranges from 1 to 17 μg/g fresh weight (FW) (Obrist et al., 2006a; Nguyen and Jehle, 2007; Székács et al., 2010). In contrast, Cry1Ab levels in pollen significantly differ among events. While concentrations in pollen from Bt176 were reported to range from 5 to 11 μg/g FW, levels in today's Bt maize events MON810 and Bt11 are much lower, i.e., in the ng/g range (Dutton et al., 2003b; Obrist et al., 2006c; Nguyen and Jehle, 2007; Li et al., 2008).

In 1998, Hilbeck et al. reported that Cry1Ab-producing Bt maize (event Bt11) and pure Cry1Ab protein harmed larvae of the green lacewing *Chrysoperla carnea* (Neuroptera: Chrysopidae) (Hilbeck et al., 1998a,b), a common predator in many crop and non-crop habitats (Box 2). These results received considerable attention in the scientific and popular press, particularly in Europe. They were the first indication of adverse effects of Lepidoptera-active Bt maize on a beneficial species unrelated to the target organisms. Those reports have been repeatedly used as evidence of environmental risks related to the use of Bt maize. Authorities in Greece (EFSA, 2006) and Austria (EFSA, 2008), for example, cited them when invoking a safeguard clause in European genetic technology legislation to suspend the approval of MON810.

Box 2 **Green lacewings.**

**Figure 1:**
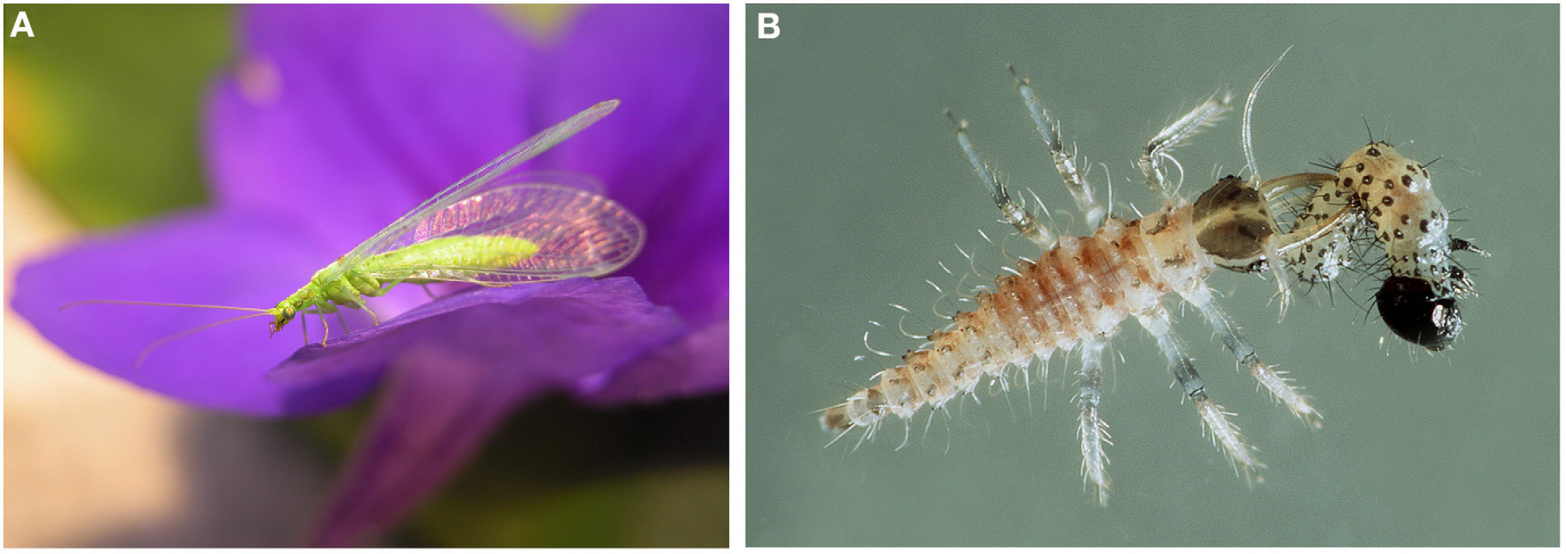
**Adult (A) and Larva (B) of the green lacewing *Chrysoperla carnea***. Photos: Agroscope (**A**, Mario Waldburger; **B**, Gabriela Brändle).

Green lacewings in the genus *Chrysoperla* (Neuroptera: Chrysopidae) are widespread in agricultural areas worldwide (Figures 1A,B). The species are valued because the larvae are predators of small soft-bodied arthropods and thus contribute to the biological control of crop pests. The adults feed predominately on pollen, nectar, and homopteran honeydew. The most common species found throughout Europe is *Chrysoperla carnea* (Duelli, 2001; Meissle et al., 2012; Romeis et al., 2014). For the purpose of this paper, the name *C. carnea* is used for the *carnea* species group that includes a complex of cryptic, sibling species that are reproductively isolated by their mating songs (Duelli, 2001; Henry et al., 2001).

Since these initial reports by Hilbeck et al. (1998a,b), numerous studies with green lacewings and Bt maize have been conducted. We here compile all available evidence regarding Bt maize effects on green lacewings. In contrast to the initial reports, we provide evidence that Bt maize producing Cry1Ab does not harm this important insect predator. We believe that the evidence available today should end the debate about Bt maize effects on green lacewings and that the case of the green lacewing can provide guidance on how findings from laboratory or field experiments with other non-target species should be placed into a risk assessment context.

## Impact of Bt maize on *C. carnea*

Two main hypotheses can be formulated on how Bt maize could cause harm to *C. carnea* and its biological control function. First, the intended production of the insecticidal Cry1Ab protein could result in direct or indirect harm. Second, the genetic modification of the maize plant could have resulted in unpredictable and unintended adverse changes.

**Figure 2:**
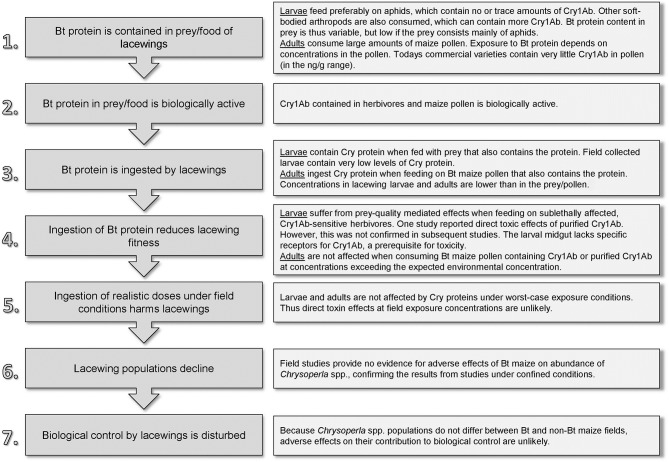
**The pathway of how Bt (Cry1Ab) maize might directly harm *Chrysoperla carnea* and a summary of published findings**.

For the assessment of potential direct toxic effects, we construct a “pathway to harm” as a conceptual model that assists in formulating and testing specific risk hypotheses (Figure 2) (Raybould, 2010; Gray, 2012). Steps 1–3 concern the Cry1Ab level that lacewings are exposed to in Bt maize fields; Steps 4 and 5 concern hazard characterization; and Steps 6 and 7 relate to the consequences of hazard and exposure. Using published data, we will discuss these steps for *C. carnea* larvae and adults. Subsequently, we also discuss the potential importance of indirect, food-web related effects and briefly address transformation related effects.

## Direct toxin effects

### Step 1. Bt protein is contained in prey/food of lacewings

Cry proteins from Bt are gut active and thus toxic only after ingestion. Step 1 assesses whether the prey/food of lacewings contains the insecticidal compound.

#### Larvae

*C. carnea* larvae are generalist predators that feed on soft-bodied arthropods but especially on aphids and other homopterans. They have been reported to feed on >70 prey species in six arthropod orders (Balduf, 1939; Herard, 1986).

Analyses of Bt maize-collected herbivores that are considered prey for *C. carnea* confirmed that they contain Cry proteins (Harwood et al., 2005; Obrist et al., 2006a; Meissle and Romeis, 2009a). Three facts are evident: (i) phloem-feeders, like aphids, contain no or only trace amounts of Cry protein when feeding on Cry1Ab-producing Bt maize (Romeis and Meissle, 2011) and Cry1Ab is not readily transported in the phloem of events Bt11 and Bt176 (Raps et al., 2001); (ii) spider mites such as *Tetranychus urticae* (Acarina: Tetranychidae) contain levels of Cry1Ab generally similar to those in green maize leaves (Dutton et al., 2002; Obrist et al., 2006b; Alvarez-Álfageme et al., 2008); (iii) Cry protein levels are considerably lower in other arthropods including tissue-feeding caterpillars and thrips than in plant tissue. While *C. carnea* larvae can utilize maize pollen and likely consume pollen during anthesis (Pilcher et al., 1997; Meissle et al., 2014), pollen in current Bt maize events contains very low levels of Cry1Ab (see below).

#### Adults

In contrast to larvae, *C. carnea* adults are not predaceous and feed mainly on pollen, nectar, and honeydew (Sheldon and MacLeod, 1971; Principi and Canard, 1984; Villenave et al., 2006; Hogervorst et al., 2007). Nectar and honeydew can be excluded as exposure routes; maize does not produce nectar, and aphid honeydew will not contain the Cry protein given that the aphids do not ingest the protein (Romeis and Meissle, 2011). Pollen, however, could expose adult *C. carnea* to Cry1Ab. Females in particular require pollen as a protein source for egg production (Li et al., 2008, 2010). Maize produces large amounts of pollen but pollen is available for ≤2 weeks in a maize field and ≤4 weeks in the surrounding landscape (Sears et al., 2001). Investigations in a flowering Swiss maize field found that all female lacewings collected during maize anthesis contained maize pollen (Li et al., 2010). Based on pollen counts in the female gut and the duration of gut passage, a female lacewing was estimated to consume up to 10,000 maize pollen grains per day at peak flowering (Li et al., 2010). While pollen of Bt maize is known to contain Cry1Ab, levels vary among events. Levels detected in MON810 and Bt11 pollen are in the ng/g range but levels more than 100-times greater than this occur in Bt176 pollen (Box 1).

### Step 2. Bt protein in prey/food is biologically active

The Cry protein contained in the food consumed by lacewings must be biologically active to cause harm.

#### Larvae

Feeding studies with sensitive caterpillars have revealed that the Cry1Ab protein in larvae of *Spodopera littoralis* (Lepidoptera: Noctuidae) or in *T. urticae* that consumed Bt maize (Bt 11) has the same biological activity as the toxin in the plant (Obrist et al., 2006b). Similarly, bioactivity of Cry proteins has been confirmed in sensitive insect bioassays for various other combinations of Bt plants, Cry proteins, and herbivores (Chen et al., 2008; Meissle and Romeis, 2009b; Li et al., 2011; Liu et al., 2011; Tian et al., 2012, 2013).

#### Adults

The biological activity of Cry1Ab in maize pollen from transformation events Bt176, Bt11, and MON810 has been confirmed in assays with sensitive insects (Hellmich et al., 2001; Dively et al., 2004; Anderson et al., 2005).

### Step 3. Bt protein is ingested by lacewings

Even though the food items consumed by lacewings contain Cry1Ab, whether the insects actually ingest the toxin when feeding should be confirmed, especially in the case of lacewing larvae. First, prey may contain Cry1Ab in their gut but the lacewing larvae may feed on the haemolymph. Second, larvae may use extra-oral digestion, albeit at a low level (Yazlovetsky, 2001), that could partially degrade the Bt protein before it enters the lacewing gut.

#### Larvae

Tri-trophic laboratory studies using *S. littoralis* larvae, *T. urticae*, and *Frankliniella tenuicornis* (Thysanoptera: Thripidae) as prey confirmed a flow of Cry1Ab from Bt maize to the herbivores and subsequently to the *C. carnea* larvae (Obrist et al., 2005, 2006b). The Cry1Ab concentration detected in *C. carnea* larvae was related to the toxin content in the prey and to the quantity of prey ingested. The Cry1Ab concentration detected in the predator was about half the concentration in the prey. This relatively small drop in concentration might be explained by the short duration of the feeding experiment and the fact that little of the ingested Cry1Ab may have been digested in the lacewing gut. That *C. carnea* larvae ingest Cry protein when feeding on Bt-containing herbivores has been reported from other tri-trophic study systems (Wei et al., 2008; Lawo et al., 2010) and another lacewing species, *Chrysoperla rufilabris* (Neuroptera: Chrysopidae) (Tian et al., 2013).

Few studies have quantified Bt Cry proteins in field-collected *C. carnea* larvae. Obrist et al. (2006a) reported levels below 0.01 μg of Cry1Ab/g dry weight (DW) in larvae collected from a Bt maize field (Bt176) in Spain before and during pollen shedding. Levels were higher (around 0.5 μg/g DW) after pollen shedding, probably because of the presence of spider mite prey that are known to contain high amounts of Cry protein (see above). No Cry1Ab was detected in two *C. carnea* samples collected in a Bt maize field (Bt11) (Harwood et al., 2005). In a Cry3Bb1-producing Bt maize field, *C. carnea* larvae contained 1.05 ± 0.932 μg of Cry3Bb1/g DW (Meissle and Romeis, 2009a). Overall, levels detected in field-collected *C. carnea* larvae were extremely low when compared to other predatory arthropods (Harwood et al., 2005; Obrist et al., 2006a; Meissle and Romeis, 2009a), probably because their main prey, i.e., aphids, do not contain Cry1Ab.

#### Adults

Li et al. (2010) quantified the amount of Cry protein that adult *C. carnea* would be exposed to when consuming only Bt maize pollen. Considering the number of pollen grains consumed by a female in a flowering maize field and the toxin concentration measured in pollen from Bt176, a female would ingest 22.6 ng of Cry1Ab per day (Li et al., 2010). The pollen grains are only partly digested during gut passage, and up to 40% of the Cry protein remains in the pollen grains that are excreted (Li et al., 2010).

Only one study has analyzed the Cry1Ab content in adult *C. carnea* collected in a Bt maize field (Bt176). Insects collected during anthesis contained about 0.5 μg/g DW (Obrist et al., 2006a). For comparison, in a Cry3Bb1-expressing Bt maize field (MON88017), adults contained 1.53 ± 0.402 μg of Cry3Bb1/g DW (Meissle and Romeis, 2009a). This difference could be explained by the substantially higher Cry protein concentrations in MON88017 compared with Bt176 pollen (Obrist et al., 2006a; Meissle and Romeis, 2009a).

### Step 4. Ingestion of Bt protein reduces lacewing fitness

Several studies assessed the toxicity of Cry1Ab to *C. carnea* larvae or adults.

#### Larvae

*C. carnea* larvae were exposed to Cry1Ab directly through artificial diet or indirectly through the provisioning of Bt maize-fed herbivores (tri-trophic studies).

#### Direct studies

Laboratory studies (Hilbeck et al., 1998a) revealed adverse effects of Cry1Ab on *C. carnea* larvae. The test compound was mixed into an artificial diet of unknown composition. When larvae were continuously fed the artificial diet containing Cry1Ab at a nominal concentration of 100 μg/ml diet, pre-imaginal mortality was 57% as compared with 30% in the untreated control. The control mortality in these studies was unusually high (Van Emden, 1999; Vogt et al., 2000), indicating problems in study design, artificial diet, or test insects (Romeis et al., 2011). In two subsequent studies, which followed a different approach, *C. carnea* larvae were provided with Cry1Ab dissolved in a sucrose solution only during the first day of each of the three larval stages (Romeis et al., 2004; Lawo and Romeis, 2008). At all other times, larvae were fed with *Ephestia kuehniella* (Lepidoptera: Pyralidae) eggs to allow their development. Consequently, larvae were not continuously exposed to Cry1Ab. To account for this, Cry1Ab was provided in high nominal concentrations of up to 0.1% Cry1Ab/ml sucrose solution (w/v). Neither study observed any adverse effects of the Cry1Ab treatment. Further evidence for a lack of effect of Cry1Ab on *C. carnea* larvae was provided by Rodrigo-Simón et al. (2006), who did not observe binding of the Cry protein to lacewing gut membranes, a prerequisite for toxicity of Bt proteins (Schnepf et al., 1998), in either histopathological or *in vitro* binding studies. Recent feeding studies with Cry1Ab containing pollen from Bt maize (Bt176, MON810) did not reveal adverse effects on the mortality or the development time of *C. carnea* larvae when compared to pollen from non-transformed near-isolines (Meissle et al., 2014). An artificial diet study also reported that Cry1Ab did not affect *C. sinica* larvae (Li et al., 2014a).

#### Tri-trophic studies

In these studies, Bt maize-fed arthropods were used to expose the lacewing larvae to Cry1Ab. Adverse effects on larval survival and development occurred when Cry1Ab-sensitive *O. nubilalis* or *S. littoralis* caterpillars were used as prey (Hilbeck et al., 1998a, 1999; Dutton et al., 2002). In contrast, *C. carnea* larvae were not affected when non-sensitive spider mites, *T. urticae*, were used as prey even though they contained about 3.5-times higher Cry1Ab concentrations than *S. littoralis* larvae (2.5 vs. 0.72 μg of Cry1Ab/g FW) (Dutton et al., 2002). Because the Cry1Ab contained in *T. urticae* and *S. littoralis* larvae is biologically active (see above), the effects observed when using Cry1Ab-sensitive caterpillars as prey were likely caused by reduced nutritional quality of sublethally affected caterpillars. Two studies provide further evidence. First, the same negative effects on *C. carnea* larvae were observed when the caterpillar prey had fed on non-transgenic plants that had been treated with a Bt spray product (Dutton et al., 2003a). Second, Lawo et al. (2010) observed adverse effects when the *C. carnea* larvae were fed with Cry1Ac-sensitive *Helicoverpa armigera* (Lepidoptera: Noctuidae) caterpillars that had consumed Bt (Cry1Ac) cotton. The predator larvae remained unaffected, however, when provided with Bt cotton-fed caterpillars from a Cry1Ac-resistant strain of *H. armigera* even though *C. carnea* larvae ingested 3.5-times more Cry1Ac when consuming resistant caterpillars than susceptible ones. These examples demonstrate that objectives must be clearly formulated and experiments carefully designed. Prey quality-mediated effects have been observed in numerous tri-trophic feeding studies with Bt-transgenic crops and a range of natural enemies (Romeis et al., 2006; Naranjo, 2009).

In summary, while early studies suggested direct toxic effects of Cry1Ab on *C. carnea* larvae, these effects were not confirmed in follow-up studies. Adverse effects in tri-trophic studies appear to be due to changes in prey quality rather than to Cry1Ab toxicity. The potential ecological relevance of such indirect Cry1Ab-related effects are discussed below.

#### Adults

Adult survival, pre-oviposition period, fecundity, fertility, and dry weight were similar when adults were fed a pure artificial diet or an artificial diet containing Cry1Ab at a nominal concentration of 120 μg/g DW (measured concentration: 47–55 μg/g DW), or when fed non-Bt maize pollen or Bt maize (Bt176) pollen containing Cry1Ab at 10 μg/g DW (Li et al., 2008). For comparison, pollen of today's Bt maize events MON810 and Bt11 contains Cry1Ab at levels that are at least 50-times lower (see above). These findings strongly suggest that *C. carnea* adults are not affected when feeding on Bt maize pollen. Similarly, Mason et al. (2008) provided no evidence for adverse effects of Cry1Ab-containing Bt maize pollen on longevity, fecundity, or fertility of adult *Chrysoperla plorabunda* (Neuroptera: Chrysopidae).

### Step 5. Ingestion of realistic doses under field conditions harms lacewings

While Steps 1–4 addressed the potential of *C. carnea* larvae and adults to be exposed to biologically active Cry1Ab in a Bt maize field and the potential sensitivity (hazard) to Cry1Ab, Step 5 assesses whether *C. carnea* larvae or adults are affected by Cry1Ab at concentrations encountered under field conditions.

#### Larvae

Under worst-case exposure conditions, only one study has suggested a direct toxic effect of Cry1Ab on *C. carnea* larvae, an effect that could not be confirmed in subsequent studies (see Step 4 above). Together with the fact that exposure of larvae is likely to be negligible through their preferred aphid prey (see Step 1), this indicates that larvae are unlikely to be affected by realistic field concentrations of Cry1Ab.

#### Adults

For adults, no sensitivity to Cry1Ab under worst-case exposure conditions has been observed (see Step 4 above). This together with the fact that the pollen from today's Bt maize events contains only low levels of Cry1Ab and that maize pollen is only available for a limited time suggests that adults are unlikely to be affected by realistic concentrations of Cry1Ab.

### Steps 6. Lacewing populations decline

Lacewing populations (*Chrysoperla* spp. or Chrysopidae incl. *Chrysoperla* spp.) in Bt (Cry1Ab) and non-Bt maize have been compared in 13 field studies. We subjected the data to a meta-analysis to determine whether lacewing abundance differs in untreated Bt maize and non-Bt maize that was treated with insecticide or left untreated (Box 3). Chrysopidae abundance did not significantly differ between Bt maize, and non-Bt maize without or with insecticide. *Chrysoperla* spp. abundance did not differ between Bt and non-Bt maize without insecticides, but was significantly higher in Bt maize without insecticides than in non-Bt maize with insecticide. Overall, the meta-analysis indicates that lacewing abundance is not reduced in Bt-maize fields. A recent meta-analysis with data from experiments with Lepidoptera-resistant Bt maize conducted in Spain has confirmed this lack of effects (Comas et al., 2014). Similar observations have been made for many other natural enemies (Wolfenbarger et al., 2008; Naranjo, 2009).

Box 3 **Evidence from field studies.**

**Figure 3:**
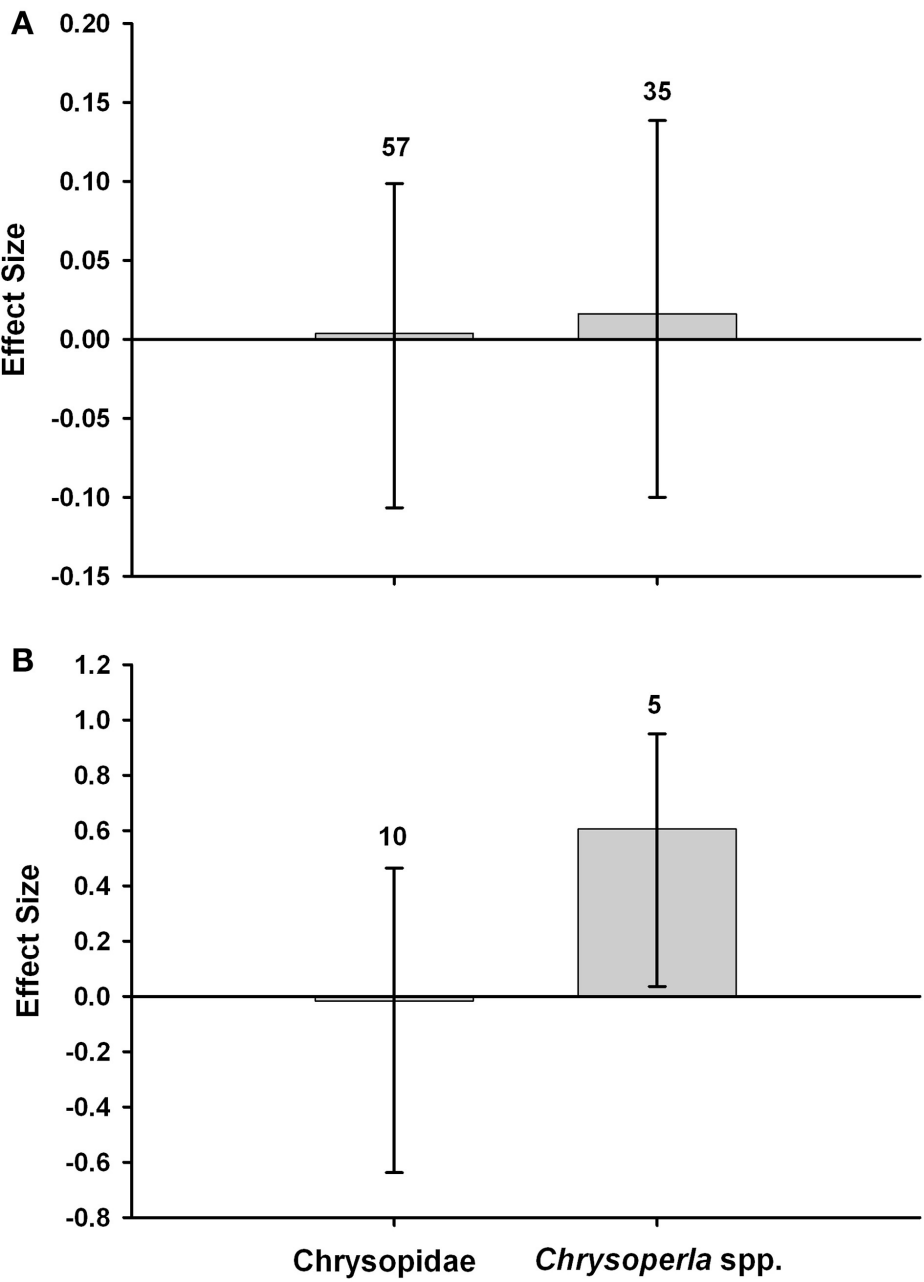
**Meta-analyses of field studies examining the abundance of lacewings in Bt maize, and non-Bt maize that was untreated with insecticide (A) or treated with insecticides (B)**.

Meta-analyses were conducted with data from field studies to compare the abundance of *Chrysoperla* spp. or Chrysopidae (incl. *Chrysoperla* spp.) in Bt (Cry1Ab) maize, and non-Bt maize that was untreated with insecticides (Figure 3A) or treated with insecticides (primarily pyrethroids) for control of the target pest (Figure 3B). The meta-analysis methodology followed the approach of Wolfenbarger et al. (2008) and Naranjo (2009). The analysis used Hedges' d, a weighted mean effect size estimator that is calculated as the difference in abundance between an experimental (Bt) and control (non-Bt) mean response divided by a pooled standard deviation and multiplied by a small sample size bias correction term. A positive effect size would indicate higher abundance in the Bt crop. In comparisons of Bt maize vs. non-Bt maize without insecticides, effect sizes were positive but they were small and did not differ from zero based on bootstrap, bias-corrected 95% confidence intervals (Figure 3A) (Rosenberg et al., 2000). In comparisons of unsprayed Bt maize vs. non-Bt maize treated with insecticides, the abundance of *Chrysoperla* spp. was significantly higher in Bt fields. The effect size for all Chrysopidae combined did not differ from zero (Figure 3B). The values above the bars denote the total number of observations. Data used for these meta-analyses are provided in Supplemental Table S1.

### Step 7. Biological control by lacewings is disturbed

Biological control by *C. carnea* or other lacewings has not been directly studied in Bt maize fields. Given a lack of population-level effects, disturbance of biological control is not expected. This is supported by evidence from Bt (Cry1Ac) cotton (Naranjo, 2005). Predation of *Bemisia tabaci* (Hemiptera: Aleyrodidae) nymphs was unchanged in Bt cotton where *Chrysoperla* spp. is an important predator (Naranjo and Ellsworth, 2005).

In addition, even if green lacewing abundance was reduced, this may not necessarily decrease biological control. For cotton, Naranjo (2005) showed that an average change of about 20% in a small number of predatory species was not ecologically meaningful in terms of the biological control potential of the natural enemy community. Furthermore, functional theory suggests that the activity of any one species in the complex can be offset by other members of the community (Walker, 1992; Naranjo, 2005).

## Indirect effects due to Cry1Ab

As discussed above, the ingestion of Cry1Ab by sensitive lepidopteran larvae (but not in the case of other non-sensitive herbivores) can reduce their nutritional quality as prey for *C. carnea* larvae and thus potentially impose adverse effects. For *C. carnea* inhabiting Bt maize fields, we regard this risk as negligible for two main reasons. First, caterpillars are generally a rare prey for lacewing larvae in Bt or non-Bt maize fields. The most common species in maize are the stem-borers (Crambidae or Noctuidae) that are the targets of Bt maize (Meissle et al., 2012; Romeis et al., 2014) and are thus virtually absent. In addition, stem-borer larvae are concealed with the stem for most of their development time and thus unavailable to predators like *C. carnea*. Larvae of other Lepidoptera species that feed on the plant surface are also relatively rare when compared to other prey and are not a preferred food source. Choice experiments have demonstrated a clear preference of *C. carnea* for aphids over *S. littoralis* larvae (Meier and Hilbeck, 2001) and behavioral observations have revealed that more than 70% of *S. littoralis* larvae that were encountered by *C. carnea* on maize plants escaped predation (Dutton et al., 2003b). In addition, *C. carnea* larvae preferred non-Bt maize-fed *S. littoralis* compared with Bt (Cry1Ab) maize-fed *S. littoralis* when given a choice (Meier and Hilbeck, 2001). Thus, exposure of *C. carnea* to sublethally affected caterpillar prey is likely to be very low. Second, available data from field experiments provide no indication that the lacewing populations decline in Bt maize fields compared with conventional maize (see Steps 6 and 7 above).

## Transformation related effects

Since the development of the first GM plants, concerns have been expressed that harm to the environment as well as harm to animal and human health could occur as a consequence of unpredictable, unintended changes that are caused by the process of transgene insertion. This process could lead to sequence changes, production of new proteins, formation of new metabolites or altered levels of existing metabolites, thus compromising safety (Cellini et al., 2004; Herman and Price, 2013).

Consequently, any new GM plant is compared to conventional counterparts, which have a history of safe use (Garcia-Alonso, 2010). The comparative assessment is based on a molecular characterization, compositional analyses, and an agronomic/phenotypic comparison. The aim of this assessment is to identify possible differences in plant characteristics (in addition to the “intended changes”) that fall outside the range of natural variation of the crop and could lead to harm. If potentially harmful unintended changes are detected, this would trigger a more detailed assessment (Romeis et al., 2008). Thus, the comparative assessment serves as a starting point to focus the environmental risk assessment process on identified stressors of concern.

All Bt maize plants that are grown today have undergone such comparative assessments. The experience with GM plants to date has shown that the transformation process is not more likely to result in significant unintended effects compared to conventional breeding techniques. There are no known cases where plants unexpectedly produce novel toxins either after genetic transformation or as a consequence of conventional breeding. In addition, each breeding process includes several screening and selection steps that identify and eliminate plants with irregular phenotypes and composition that could cause an impact on non-target organisms (Weber et al., 2012; Herman and Price, 2013).

Surprisingly, some authors have reported an increase in aphid abundance in Bt (Cry1Ab) maize in Spain (Lumbierres et al., 2004; Pons et al., 2005). The factors that caused this phenomenon have not been elucidated but could be due to some unintended, transformation related effects. An increase in aphid abundance, however, is not expected to adversely affect lacewing abundance given that aphids are the preferred prey for lacewing larvae.

In summary, it is unlikely that unintended, transformation related effects could result in adverse effects on lacewings. This is supported by the fact that no adverse effects of Bt maize on the abundance of lacewings and biological control have been reported from the field (see Steps 6 and 7 above).

## Conclusions

Because putative adverse non-target effects of Cry1Ab Bt maize on the green lacewing *C. carnea* were reported shortly after the commercial release of this GM plant, the results have received considerable attention. In this comprehensive review, we show that there is sufficient information available today to conclude that Bt maize containing Cry1Ab does not harm *C. carnea*. Similarly, there is no evidence that other Lepidoptera-active Cry1 and Cry2 proteins produced by GM maize and other plants cause direct toxic effects to *Chrysoperla* species (OECD, 2007; Mason et al., 2008; Tian et al., 2013; Li et al., 2013, 2014a,b). Despite this, the myth that Bt maize harms green lacewings continues to be used by opponents of GM plants, who selectively cite only those initial studies that indicated direct adverse effects (Hilbeck et al., 2008, 2012; Dolezel et al., 2009; Then, 2010; Meyer, 2011).

We believe that lessons learned from the lacewing case can improve the quality and predictability of non-target risk assessment of future insecticidal GM crops. First, scientific studies can sometimes lead to unexpected results, which should be verified by other independent research groups. As was the case with *C. carnea*, harmful effects of Cry1Ab on a few other non-Lepidoptera species have been observed (Romeis et al., 2013). However, in none of the cases have the results been confirmed in follow-up studies.

Second, effects observed under laboratory conditions cannot automatically be translated into environmental harm. The development of conceptual models on how a particular GM plant could harm a valued non-target species or its ecological function assists in this process and also helps in formulating testable risk hypotheses. If studies provide convincing evidence that one of the steps in the conceptual model is unlikely, the pathway to harm is interrupted, and a negligible risk can be inferred. This requires, however, that the studies conducted are properly designed and reproducible to minimize the probability of false positives and negatives.

### Conflict of interest statement

The authors declare that the research was conducted in the absence of any commercial or financial relationships that could be construed as a potential conflict of interest.
